# Supplementary material for: Inference of long-range cell-cell force transmission from ECM remodeling fluctuations
Source: Commun Biol. 2023 Aug 3;6:811. doi: 10.1038/s42003-023-05179-1 (PMC10400639; doi:10.1038/s42003-023-05179-1)
Supplement: Supplementary file 2 — Supplementary Figures [file 42003_2023_5179_MOESM2_ESM.pdf]

Supplementary Figures

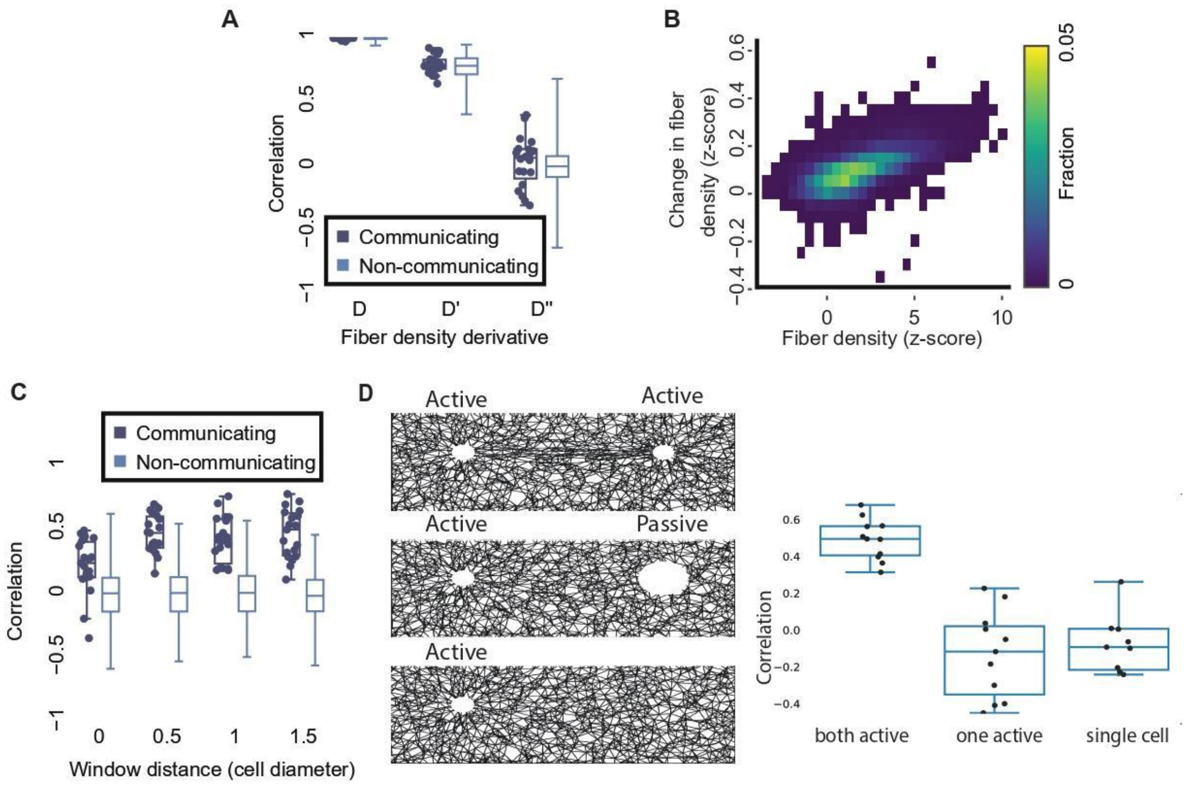

Figure S1. Temporal correlations of ECM remodeling fluctuations in communicating and non-communicating simulated cell pairs. In these simulations each cell draws its contraction in each step independently from a normal distribution with mean of 1% and standard deviation of 0.5%. (**A**) Correlations for fiber density (D), first temporal derivative (D’) and second temporal derivative (D’’) for communicating and non-communicating cell pairs. N = 20 cell pairs at a pair distance of 5 cell diameters. All p-values not significant. (**B**) Two-dimensional distribution of fiber density and change in fiber density measured in consecutive contraction steps. Quantification windows adjacent to each cell along the band. N = 120 cell pairs (N = 20 for distances of 5, 7, 9, 12, 15 and 17 cell diameters). Constant cell contraction of 1% between consecutive time frames. Pearson correlation coefficient = 0.59, p-value < 0.0001. (**C**) Correlation of communicating versus non-communicating cell pairs using the second derivative of fiber density dynamics as a function of the window distance. Pair distance = 5 (N = 19 pairs). All p-values < 0.0001. Data points for non-communicating cell pairs were not displayed in panels A and C because there are too many of them, while the number of data points for communicating cells is much smaller and thus can be plotted. (**D**) (Left) visualization of representative simulated cell-ECM interactions. Top: both cells actively contract. Middle: one cell actively contracts, another cell is fixed. Bottom: only one cell is present and active. (Right) Correlation measurements. Left: correlation of communicating cell pairs at distance 4 cell diameters, i.e., both cells actively contract. Middle: correlation of cell pairs where one, but not the other, is actively contracting. Right: correlation of a simulated single cell without a communication partner. A quantification window was placed at the same distance as the second cell would have been in the other simulations. N = 11 independent simulations per simulation condition.

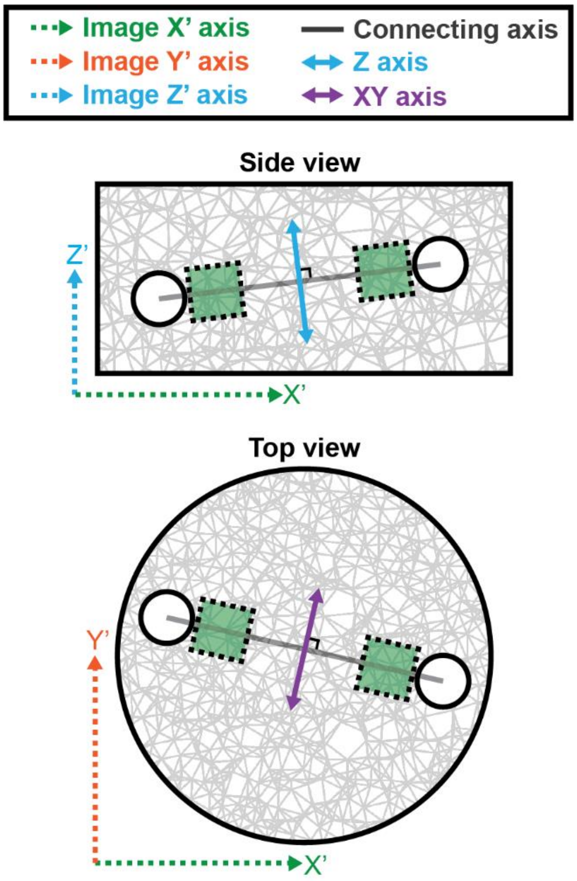

Figure S2. ECM remodeling dynamics are quantified along the connecting axis between the cells: illustration of the side view (top) and top view (bottom). Microscopy axes (X', Y', Z') marked with dashed arrows, transformed visualization and quantification axes (connecting axis, XY, Z) marked with solid arrows. The cuboid region in-between the cell pair (brown), has the width of a cell diameter, and is used for quantification along the connecting axis. The length of the cuboid region is the pair distance. The cuboid left and right sides were parallel to the microscopy axial plane. The Z-axis (cyan) and the XY-axis (purple) are perpendicular to each other and to the connecting axis (black).

A

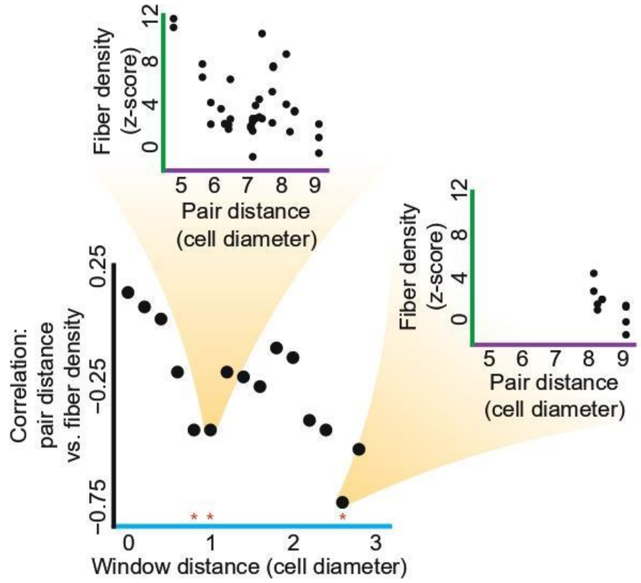

B

Window distance (cell diameter)	Correlation: pair distance vs. fiber density	N	P-value
0	0.18	54	0.21
0.2	0.1	52	0.48
0.4	0.05	49	0.73
0.6	-0.16	41	0.31
**0.8**	**-0.37**	**40**	**0.02** *
**1**	**-0.37**	**38**	**0.02** *
1.2	-0.18	35	0.3
1.4	-0.19	35	0.27
1.6	-0.23	29	0.22
1.8	-0.07	25	0.73
2	-0.11	21	0.64
2.2	-0.37	17	0.14
2.4	-0.41	14	0.14
**2.6**	**-0.71**	**10**	**0.02** *
2.8	-0.49	6	0.32

Figure S3. The distance between pairs of communicating cells is negatively correlated to the density of fibrous regions further away from the cell. I.e., as the quantification region moves away from one cell, the effect of the other cell drops as a function of the pair distance, implying a stronger mechanical interaction with the other cell for closer pairs. (**A**) Correlation between the window distance and the correlation between pair distance and fiber density across cell pairs. N = 15 window distances. Pearson correlation coefficient = -0.76, p-value < 0.001. Red asterisks reflect a significance level below 0.05. Insets: two significant correlations between fiber density pair distance for window distance of 1 (top) and 2.6 (right) cell diameters. N = 38 (top) and 10 (right) communicating cell pairs. Pearson correlation coefficient = -0.37 (top) and -0.71 (right), p-value = 0.02 for both. The reduced number of cell pairs (right) stems from overlapping of the quantification regions due to short pair distances. Note that the color coding (purple, cyan, green) is matching the sketch in panel A. (**B**) Summary table for the data in panel B. Bold rows with red asterisk reflect correlations with significance levels below 0.05.

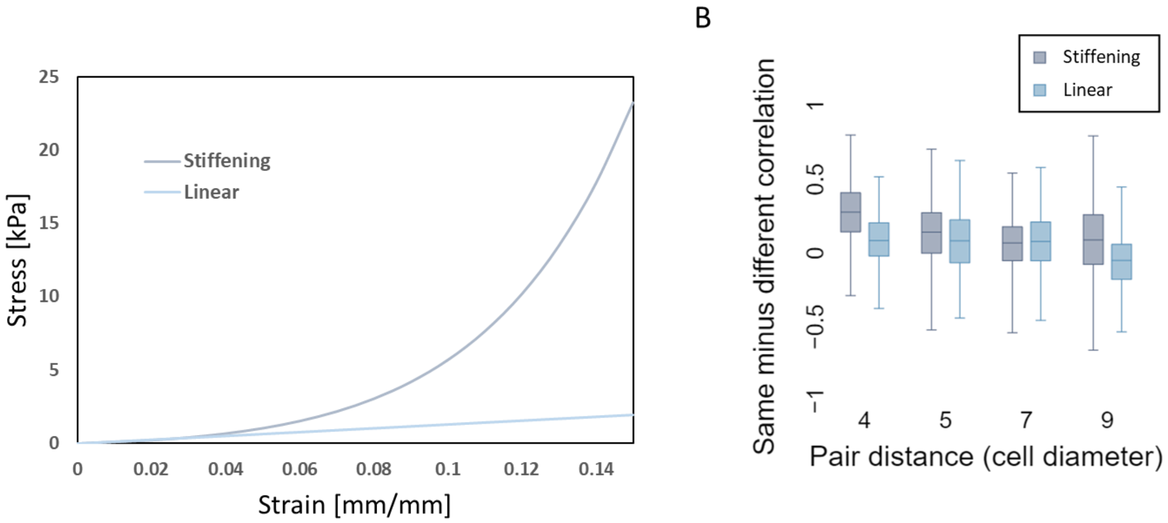

Figure S4. Comparison of fibrous material models with linear and stiffening behavior. (**A**) Stress-strain curves representing the mechanical behavior of the fibers used in the simulations. Stiffening model is based on 2.4 mg/ml collagen, linear model is based on 4 mg/ml fibrin [54]. (**B**) Same network correlations minus different network correlations as a function of the cell-to-cell pair distance (second derivative of fiber density, cell heterogeneity of mean contraction of 1% and standard deviation of 0.5%). Correlations were calculated for cell-to-cell pair distances of 4 (20 pairs for the stiffening model and 10 pairs for linear model), 5 (19 pairs for the stiffening model and 10 pairs for the linear model), 7 (19 pairs for the stiffening and 10 pairs for the linear), and 9 (19 pairs for the stiffening model and 10 pairs for the linear model) cell diameters. For ‘stiffening’, “same” > “different” in 72% of the matched same-versus-different pair analysis (92%, 79%, 70% and 68% for pair distances of 4,5,7 and 9 cell diameters, correspondingly), whereas in for ‘linear’, “same” > “different” in 61% of the matched same-versus-different pair analysis (76%, 68%, 71% and 43% for pair distances of 4,5,7 and 9 cell diameters, correspondingly).

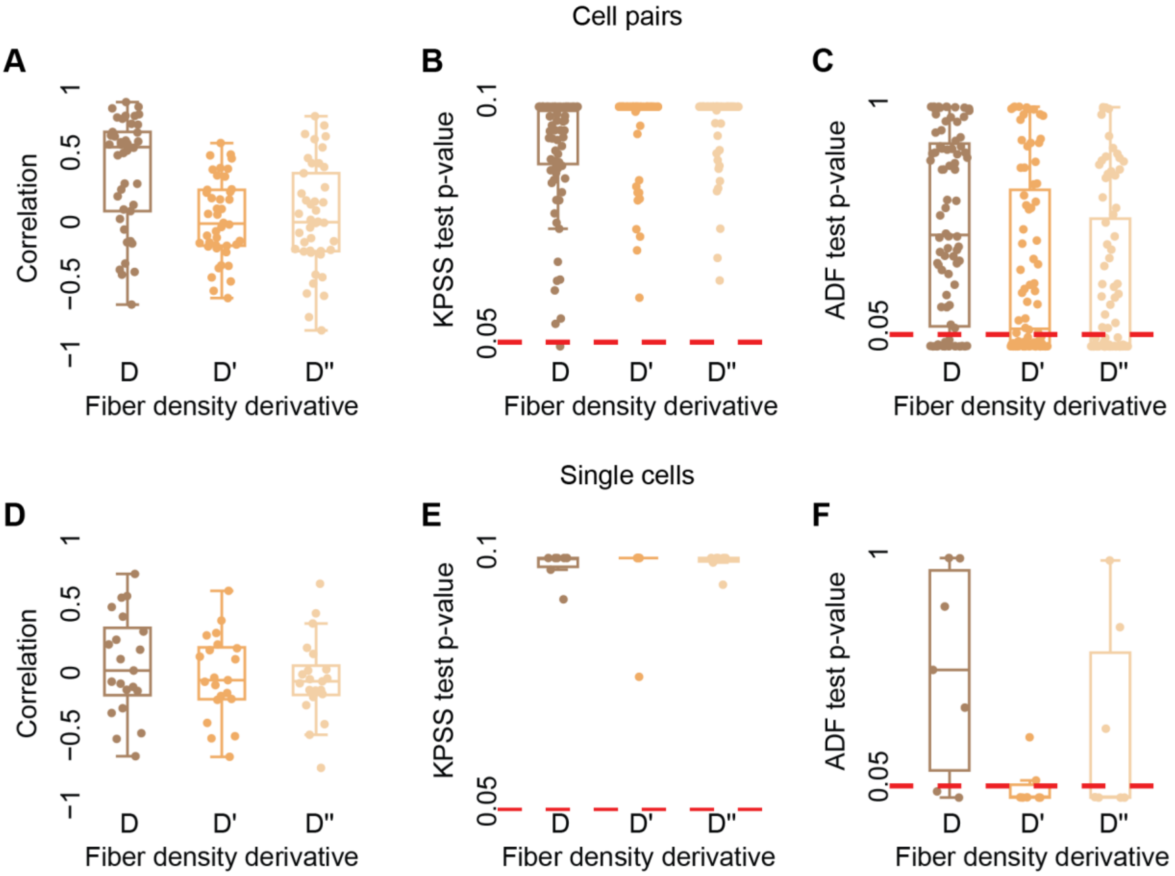

Figure S5. Detrending fiber density of single cell and cell pairs. Shown are correlations between fiber density (D), first temporal derivative (D') and second temporal derivative (D'') in time. (**A-C**) Cell pairs. Pairs distance 60-150 µm (4-10 cell diameters). N = 42 cell pairs. (**A**) Wilcoxon signed-rank testing the null hypothesis that the correlations are distributed around a mean = 0: p-value < 0.0001 (D), not significant (D', D''). (**B**) Kwiatkowski–Phillips–Schmidt–Shin (KPSS) testing the null hypothesis that the data is stationary. Each data point is the KPSS test p-value rejecting the null hypothesis that the time series is stationary, with p-value < 0.05. The null hypothesis is rejected for 99% of the raw and for 100% of first or second derivative time series. (**C**) Augmented Dickey Fuller test (ADF) testing the null hypothesis that the data is non-stationary. Each data point is the ADF test p-value rejecting the null hypothesis that the time series is not stationary, with p-value < 0.05. The null hypothesis is rejected for 24% of the raw time series, 46% for the first derivative and 55% for the second derivative. (**D-F**) Single cells. N = 7 cells. Correlations are computed between all possible pairs of single cells, total of 21 correlations. (**D**) Wilcoxon signed-rank p-value not significant (D, D', D''). (**E**) KPSS is not rejected for all raw, first, or second derivative time series. (**F**) ADF is rejected for 29% of the raw time series, 71% for the first derivative and 57% for the second derivative.

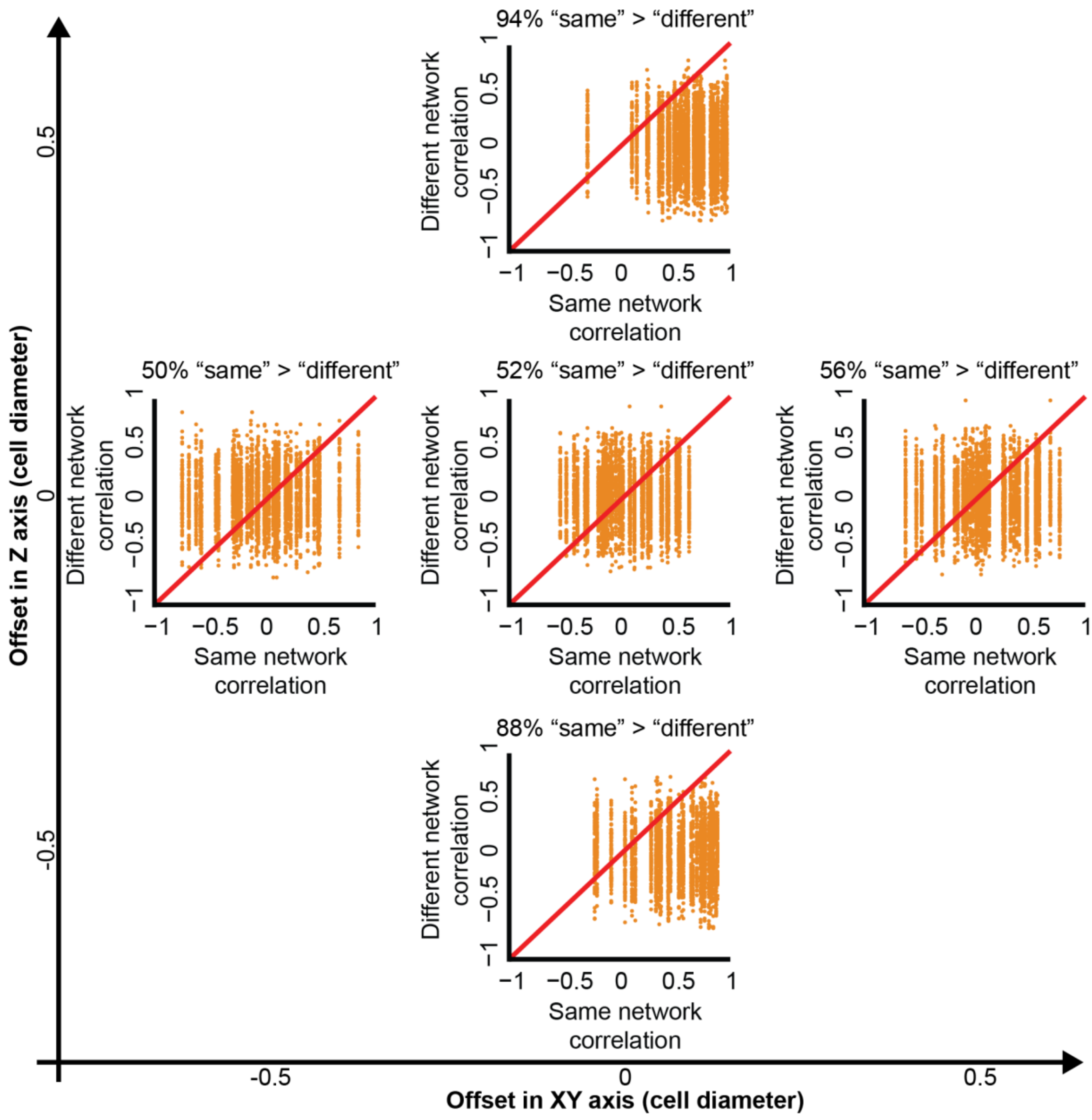

Figure S6. Enhanced identification of communicating cells by shifting the quantification window's location. Same-versus-different pair analysis for different locations of the quantification window. Pair distance was 60-150 μm (~4-10) cell diameters, window distance = 0, correlations were calculated using the first derivative of fiber density dynamics. All combinations of “same”/”different” were considered. Slightly different numbers of cell pairs per quantification window location stems from discarding pairs where the quantification windows exceeded beyond the image boundaries. See Fig. 7A for axes schematics. **Top panel (Z = 0.5, XY = 0)**: N = 48 cell pairs. “Same” pair had a higher correlation than “different” pair in 94% of the matched correlations. Wilcoxon signed-rank p-value < 0.0001. **Left panel (Z = 0, XY = -0.5)**: N = 42 cell pairs. “Same” pair had a higher correlation than “different” pair in 50% of the matched correlations. Wilcoxon signed-rank p-value not significant. **Middle panel (Z = 0, XY = 0)**: N = 42 cell pairs. “Same” pair had a higher correlation than “different” pair in 52% of the matched correlations. Wilcoxon signed-rank p-value < 0.001. **Right panel (Z = 0, XY = 0.5)**: N = 42 cell pairs. “Same” pair had a higher correlation than “different” pair in 56% of the matched correlations. Wilcoxon signed-rank p-value < 0.0001. **Bottom panel (Z = -0.5, XY = 0)**: N = 36 cell pairs. “Same” pair had a higher correlation than “different” pair in 88% of the matched correlations. Wilcoxon signed-rank p-value < 0.0001.

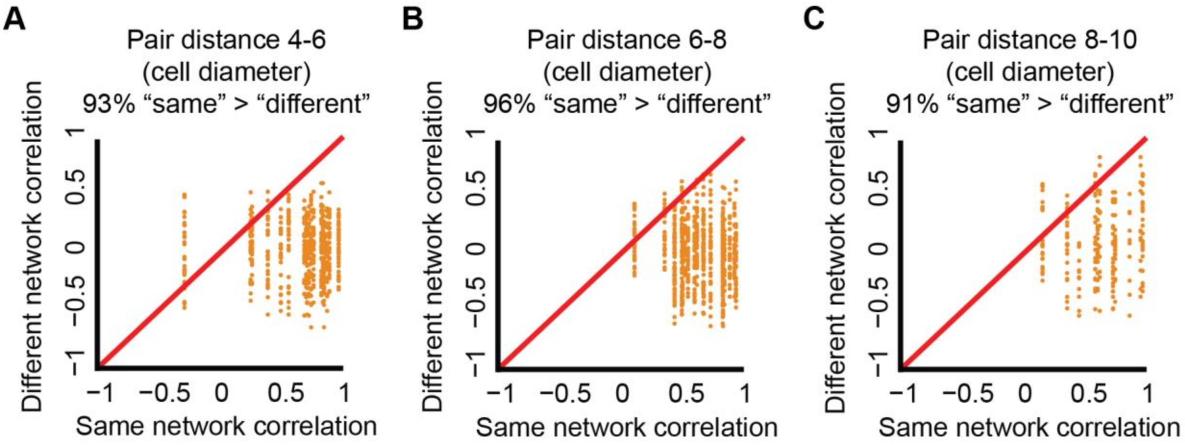

Figure S7. Identification of communicating cells in different pair distances. Same-versus-different pair analysis. Quantification windows were placed 7.5 µm above the connecting axis between the cells (see Fig. 5E for schematics). Correlations were calculated using the first derivative of fiber density dynamics. All combinations of "same"/"different" were considered. Partitioning the data to cell pairs intervals of 4-6, 6-8, or 8-10 cell diameters was performed under the assumption of a mean fibroblast diameter of 15 µm. (**A**) Pair distance 60-90 µm (4-6 cell diameters). N = 18 cell pairs. “Same” pair had a higher correlation than “different” pair in 93% of the matched correlations. Wilcoxon signed-rank p-value < 0.0001. (**B**) Pair distance 90-120 µm (6-8 cell diameters). N = 19 cell pairs. “Same” pair had a higher correlation than “different” pair in 96% of the matched correlations. Wilcoxon signed-rank p-value < 0.0001. (**C**) Pair distance 120-150 µm (8-10). N = 11 cell pairs. “Same” pair had a higher correlation than “different” pair in 91% of the matched correlations. Wilcoxon signed-rank p-value < 0.0001.

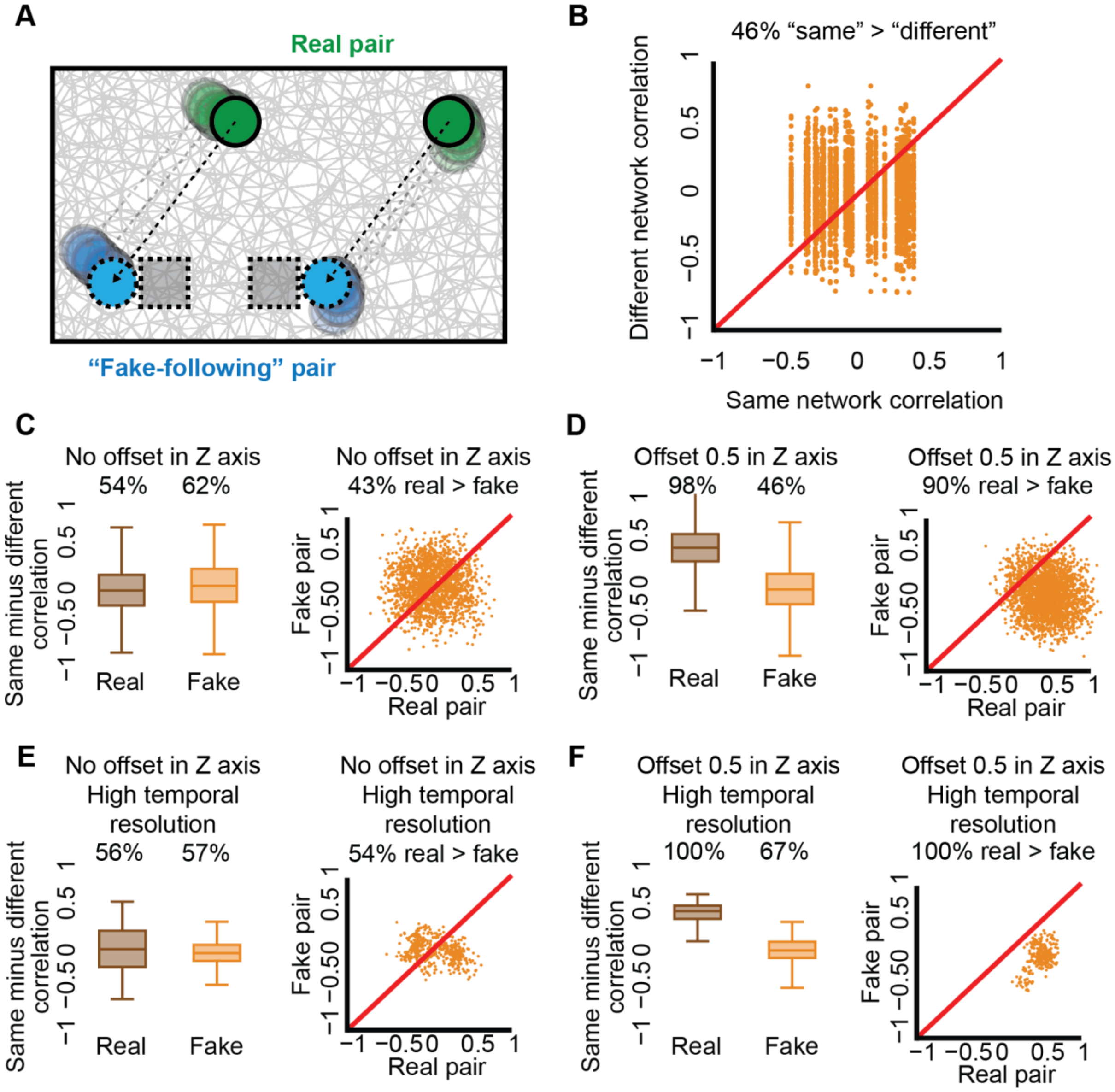

Figure S8. Controlling for potential masking of cell-ECM-cell communication by local ECM remodeling fluctuations. (**A**) Schematic sketch of a “fake-following” pair (cyan) mimicking a real cell pair (green) by repeating the cells’ shifts in X and Y axes (green shadow) in a fixed distance from the real pair (cyan shadow). (**B**) Quantification of same-versus-different analysis for “fake-following” pairs. N = 25 fake cell pairs. “Same” pair had a higher correlation than “different” pair in 46% of the matched correlations. Wilcoxon signed-rank p-value < 0.0001. (**C-F**) Cell-ECM-cell communication is more prominent than the masking by local ECM remodeling fluctuations. Same-vs-different pair analysis of cell pairs and their corresponding “fake-follower” cell pair. Pair distance 60-150 μm, correlations were calculated using the first derivative of fiber density dynamics. All combinations of "same"/"different" were considered for both the cell pairs and the “fake” cell pairs independently. Left panels compare the distribution of same-versus-different pair analysis of cell pairs (“real”) versus “fake-follower” pairs (“fake”). Right panels compare matched same-vs-different pair analysis for each cell pair (“real”) and its corresponding “fake-follower” pair (“fake”). Panels C-D and E-F are the same analysis with low (15 minutes) versus high (5 minutes) temporal resolution correspondingly. In panels C and E the quantification window is located at the focal plane between the cell pair, while in panels D and F 7.5 μm above the focal plain between the cell pair. (**C**) Quantification window at the focal plane between the cell pair. Temporal resolution = 15 minutes. N = 20 matched pairs. Left:

“same” > “different” in 54% (“real”) and 62% (“fake”) of the same-versus-different pair analysis. Wilcoxon signed-rank p-values < 0.0001. Right: “real” > “fake” in 43% of the matched same-versus-different pair analysis. Wilcoxon signed-rank p-value < 0.0001. (**D**) Quantification window 7.5 μm above focal plane between the cell pair. Temporal resolution = 15 minutes. N = 25 matched pairs. Left: “same” > “different” in 98% (“real”) and 46% (“fake”) of the same-versus-different pair analysis. Wilcoxon signed-rank p-values < 0.0001. Right: “real” > “fake” in 90% of the matched same-versus-different pair analysis. Wilcoxon signed-rank p-value < 0.0001. (**E**) Quantification window at the focal plane between the cell pair. Temporal resolution = 5 minutes. N = 19 matched pairs. Left: “same” > “different” in 56% (“real”) and 57% (“fake”) of the same-vs-different pair analysis. Wilcoxon signed-rank p-values < 0.001. Right: “real” > “fake” in 54% of the matched same-vs-different pair analysis observations. Wilcoxon signed-rank p-value < 0.05. (**F**) Quantification window 7.5 μm above focal plane between the cell pair. Temporal resolution = 5 minutes. N = 14 matched pairs. (**G**) “Same” > “different” in 100% (“real”) and 67% (“fake”) of the same-versus-different pair analysis. Wilcoxon signed-rank p-values < 0.0001. (**H**) “Real” > “fake” in 100% of the matched same-vs-different pair analysis observations. Wilcoxon signed-rank p-value < 0.0001.

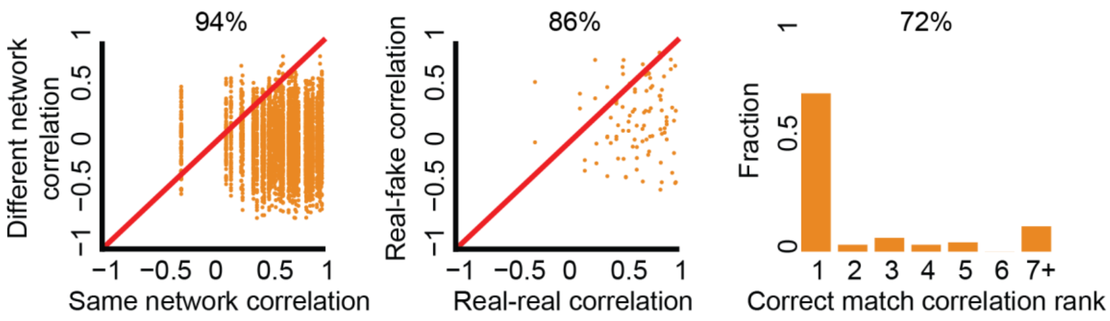

Figure S9. Cell-ECM-cell communication in Murine cancer Hras mutated cells. Same-versus-different: N = 48, 94% “same” > “different, p-value < 0.0001. Real-versus-fake: N = 48, 86% “real” > “fake”, p-value < 0.0001. Matchmaking analysis: N = 96, correct matching probability = 72%.

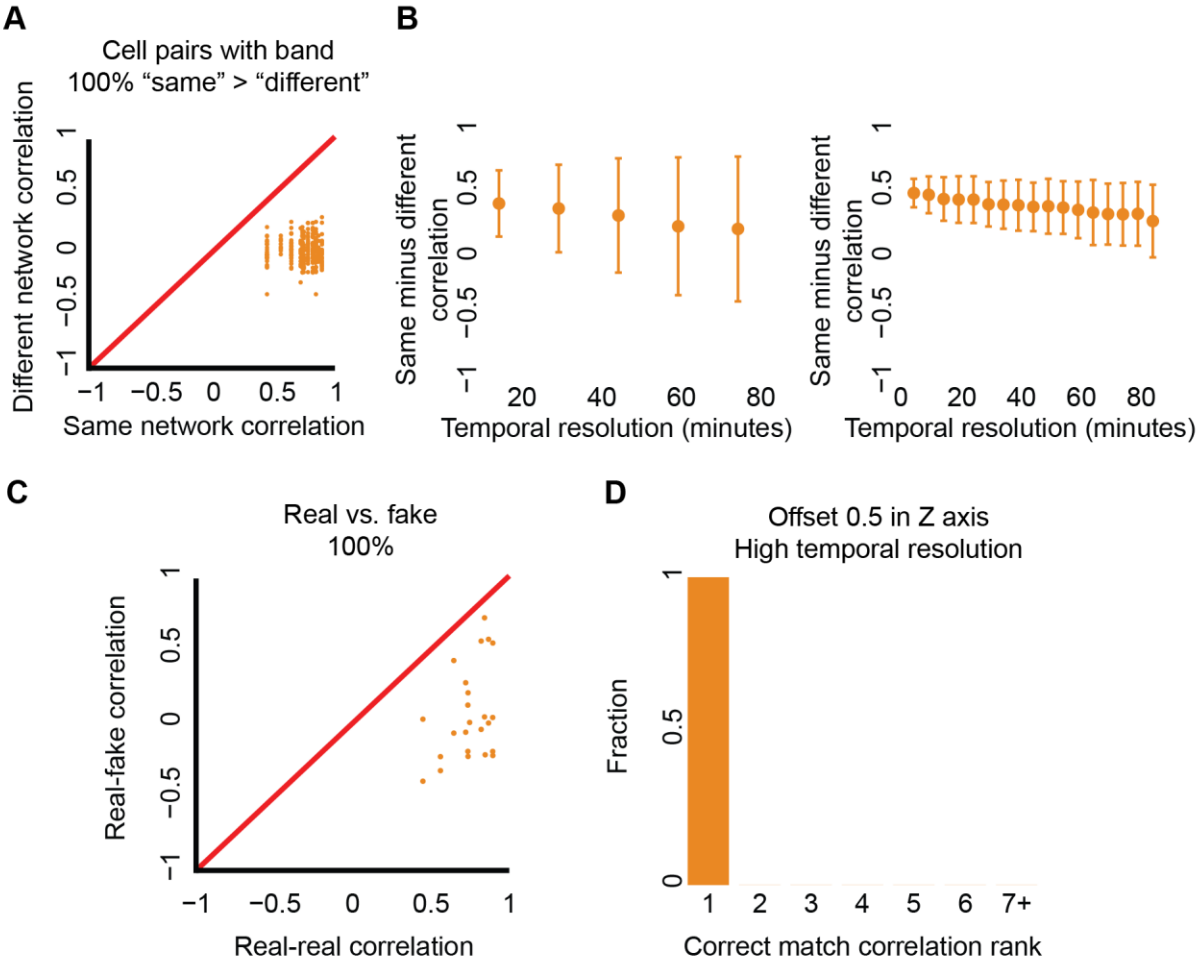

Figure S10. Sensitivity of same-versus-different pair analysis to the temporal resolution. Pair distance 60-150 µm, quantification windows were placed 7.5 µm above the connecting axis between the cells (see Fig. 7E for schematics). Correlations were calculated using the first derivative of fiber density dynamics. (**A**) Identification of communicating cells in higher temporal resolution of 5 minutes per frame. Same-versus-different pair analysis. All combinations of "same"/"different" were considered. Pairs with a visible band. N = 14 cell pairs. “Same” pair had a higher correlation than “different” pair in all (100%) of the matched correlations. Wilcoxon signed-rank p-value < 0.0001. (**B**) Sensitivity of same-versus-different pair analysis to the temporal resolution. The experimental temporal resolution was artificially down-sampled to create multiple time series with reduced time resolutions with different starting times. All possible time intervals under a given temporal resolution (different starting time) were considered leading to multiple recorded observations for a single pair of communicating cells (Methods). Left: Low temporal resolution experiments. Original time resolution was 15 minutes per frame. N = 2 experiments, same-versus-different pair analysis was performed independently for each experiment and the results were pooled together without mixing pairs from different experiments. N = 48 pairs (15/30/45 minutes), N = 43 pairs (60 minutes) and N = 14 pairs (75 minutes). “Same” pair had a higher correlation than “different” pair in 94% (15 minutes), 87% (30 minutes), 77% (45 minutes), 68% (60 minutes) and 67% (75 minutes) of the matched correlations. Wilcoxon signed-rank p-value < 0.0001 for all temporal resolutions. Right: High temporal resolution experiments (5 minutes per frame). N = 3 experiments, same-versus-different pair analysis was performed independently for each experiment and the results were pooled together without mixing pairs from different experiments. N total pairs = 14 pairs (5/10/15 minutes, 100/99/99% “same” > different” correspondingly), N = 13 pairs (20 minutes, 99% “same” > different”), N = 12 pairs (25 minutes, 99% “same” > different”), N = 9 pairs (30/35/40 minutes, 99/98/98% “same” > different” correspondingly), N = 8 pairs (45/50/55 minutes, 98/96/97% correspondingly), N = 7 pairs (60/65/70/75 minutes, 95/92/92/91% “same” > different” correspondingly), n = 6 pairs (80/85

minutes, 91/86% “same” > different” correspondingly). (**C**) Real-versus-fake for high temporal resolution: N = 13, 100% “real” > “fake”, p-value < 0.0001. (**D**) Matchmaking analysis for high temporal resolution: N = 28 cells, correct matching probability = 100%.

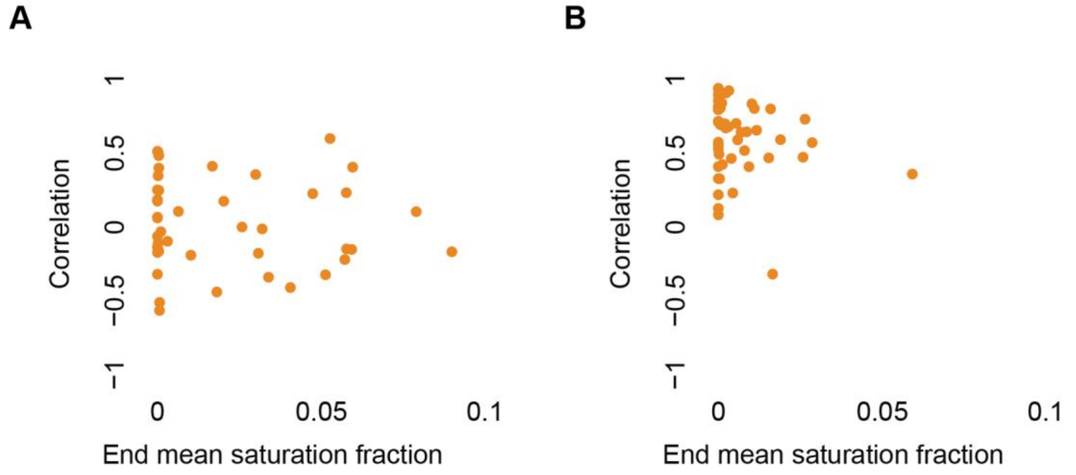

Figure S11. Association between cell pair correlations and the fraction of saturated pixels in the region used to measure cell-cell communication. For both panels, pair distances range between 4 and 10 cell diameters (60-150 μm, assuming mean fibroblast diameter of 15 μm). Correlations were calculated using the first derivative of fiber density dynamics. (**A**) Saturation-independent correlations along the connecting axis between the cells (Pearson correlation coefficient p-value > 0.05), N = 42 cell pairs. Image saturation does not hamper the ability to identify cell-ECM-cell communication along the dense fibrous band. (**B**) Image saturation slightly reduces the ability to identify cell-ECM-cell communication 0.5 cell diameters (7.5 μm) above the connecting axis between the cells. N = 48 cell pairs, Pearson correlation coefficient = -0.18, p-value < 0.0001.

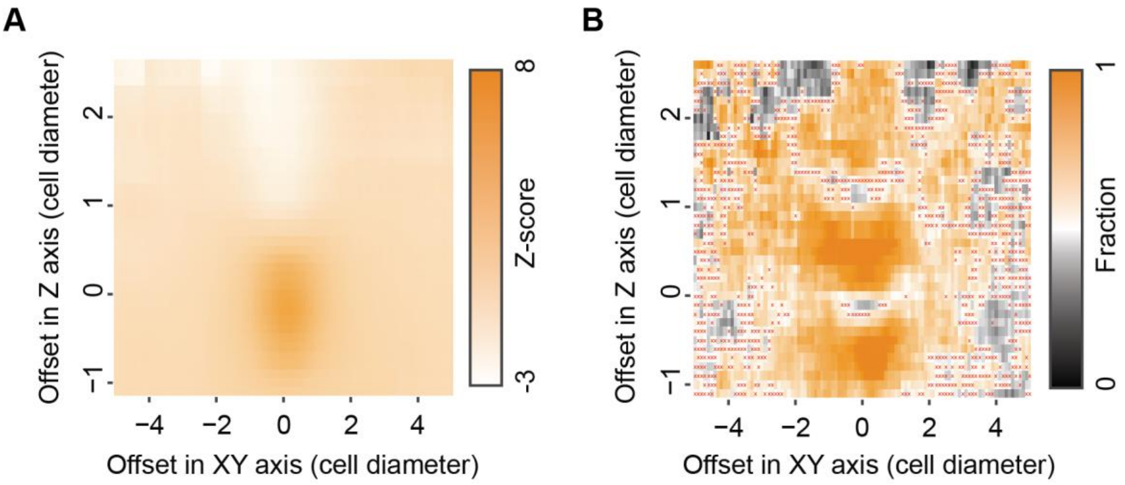

Figure S12. Spatial decoupling of band formation and cell-ECM-cell communication in cell pairs with a visible band imaged in high temporal resolution of 5 minutes per frame. Pair distance of 60-150 μm. (**A**) Fiber density. Mean fiber density for systematic offsets in Z and XY axes. (**B**) Cell-ECM-cell communication. Mean fraction of higher “same”, correlation between communicating pairs, versus “different”, correlation between one cell from that pair and another cell from a different communicating pair for systematic offsets in Z and XY axes. Red ‘x’ marked that the null hypothesis that “same” - “different” correlations are distributed around zero was not rejected with p-value ≤ 0.05.

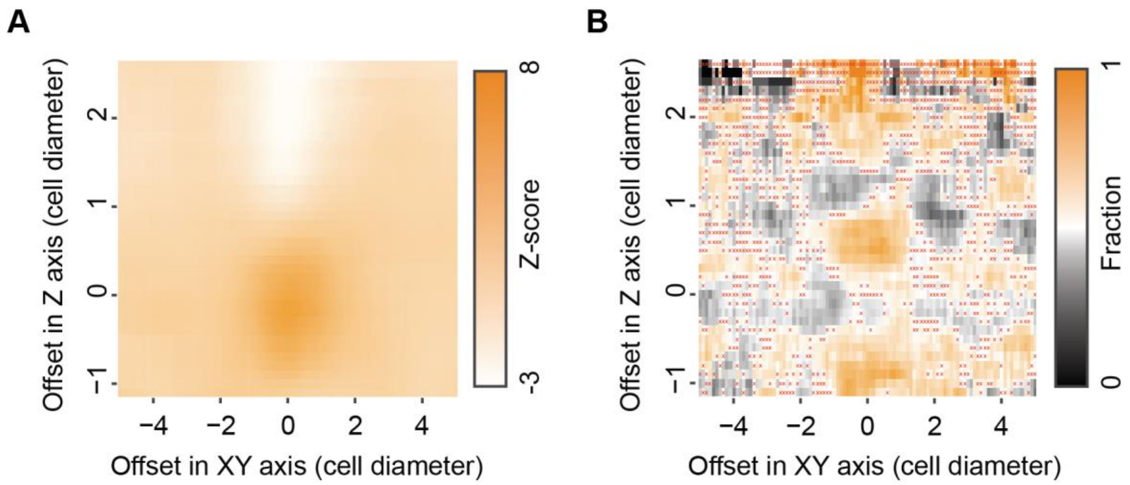

Figure S13. Spatial decoupling of band formation and cell-ECM-cell communication in pairs with no visible band imaged in high temporal resolution of 5 minutes. Pair distance of 60-150 μm. (**A**) Fiber density. Mean fiber density for systematic offsets in Z and XY axes. (**B**) Cell-ECM-cell communication. Mean fraction of higher "same", correlation between communicating pairs, versus "different", correlation between one cell from that pair and another cell from a different communicating pair for systematic offsets in Z and XY axes. Red 'x' marked that the null hypothesis that "Same" - "different" correlations are distributed around zero was not rejected with p-value ≤ 0.05.

A

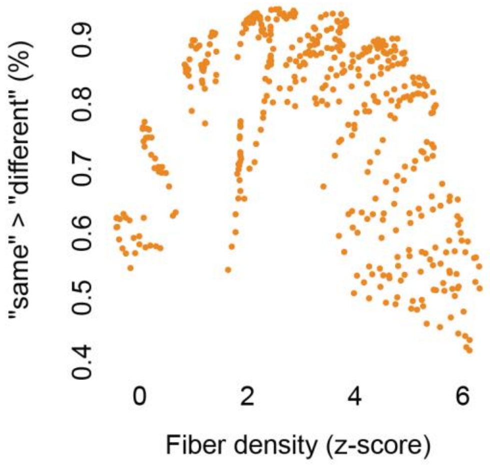

B

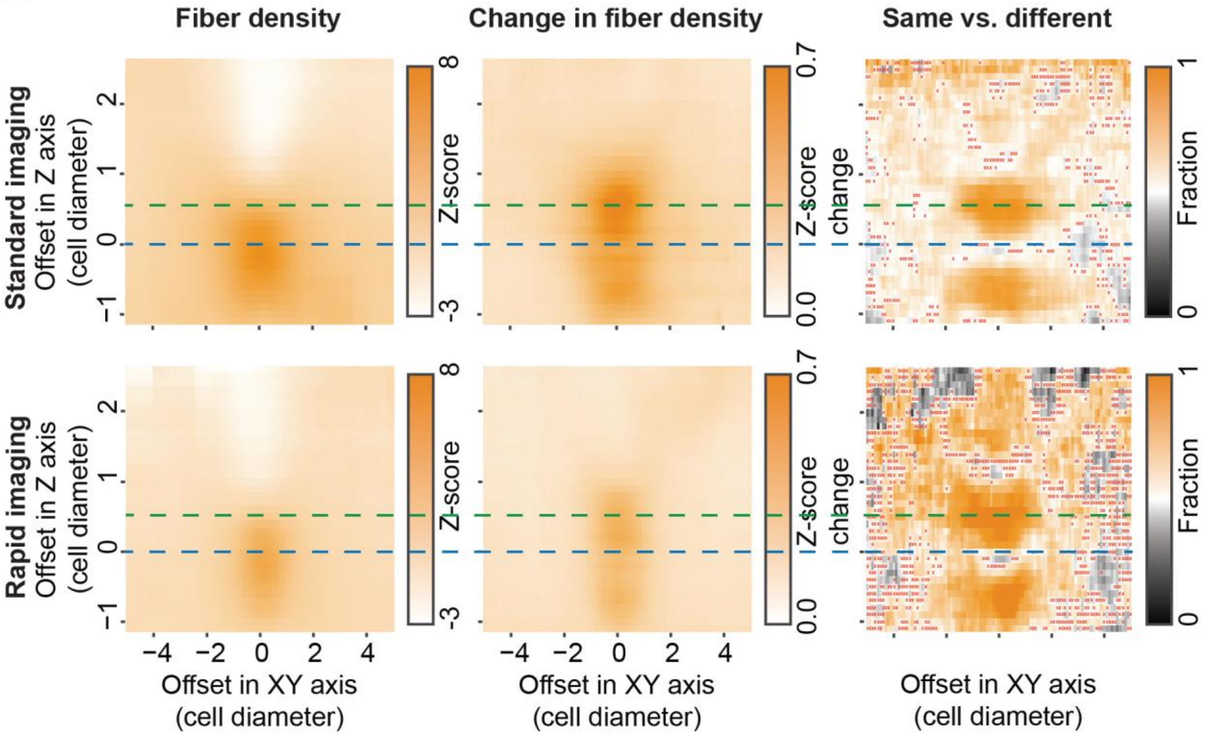

Figure S14. Higher ECM intensities along the fibrous band confound our ability to sensitively measure the ECM fluctuations as a signature of cell-ECM-cell communication. (**A**) Association between fiber density and our ability to identify cell-ECM-cell communication with same-versus-different pair analysis. Each data point represents the mean fiber density in the quantification window (x-axis) and the fraction of cell pairs with “same” correlation higher than “different” correlation (y-axis) for different locations of the quantification window. The data here was derived from matched bins in Fig. 7B (x-axis) and Fig. 7C (y-axis) in a region that spans 1 cell diameter away (15 µm) from the quantification plane defined adjacent to the cells in the connecting axis between the communicating cells: all bins in the range $-1 \le XY \le 1$ and $-1 \le Z \le 1$. Pearson correlation coefficient = -0.26, p-value < 0.0001. Note the stiff drop in cell-ECM-cell communication around fiber density of ~3-4 z-scores. (**B**) The band intensity is maximal along the connecting axis between the cells. The

magnitude of the changes in ECM intensity is similar along the connecting axis and slightly above it, where communication is optimal. Top: imaging at temporal resolution of 15 minutes per frame (left-most and right most are duplication of Fig. 7B and Fig. 7C correspondingly), bottom: imaging at temporal resolution of 5 minutes per frame (left-most and right most are duplication of Fig. S12A and Fig. S12B correspondingly).

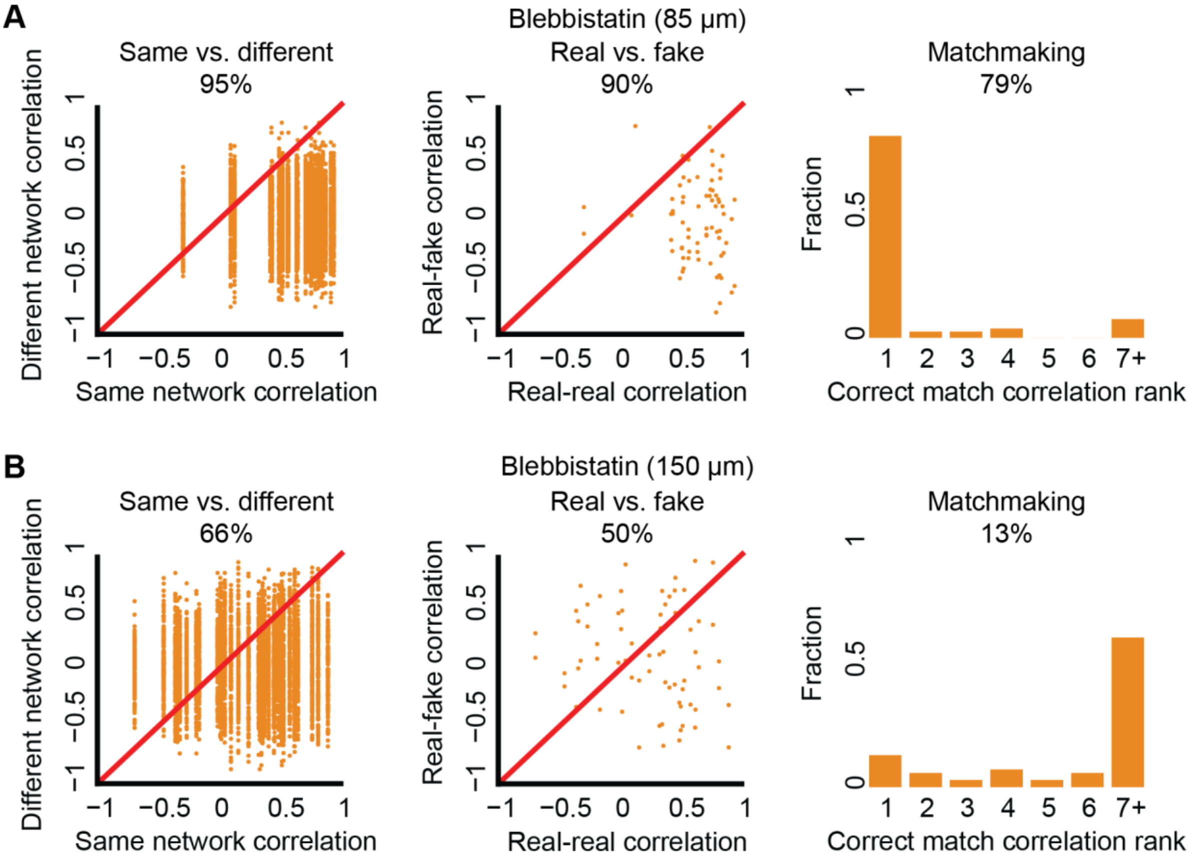

Figure S15. Contractility inhibition experiments measured with same-versus-different analysis (first column), real-versus-fake analysis (middle column), and match making analysis (right column). (**A**) 85 nM Blebbistatin. Same-versus-different: N = 42, 95% “same” > “different, p-value < 0.0001. Real-versus-fake: N = 42, 90% “real” > “fake”, p-value < 0.0001. Matchmaking analysis: N = 84, correct matching probability = 79%. (**B**) 150 nM Blebbistatin. Same-versus-different: N = 34, 66% “same” > “different, p-value < 0.0001. Real-versus-fake: N = 34, 50% “real” > “fake”, p-value not significant. Matchmaking analysis: N = 68, correct matching probability = 13%.

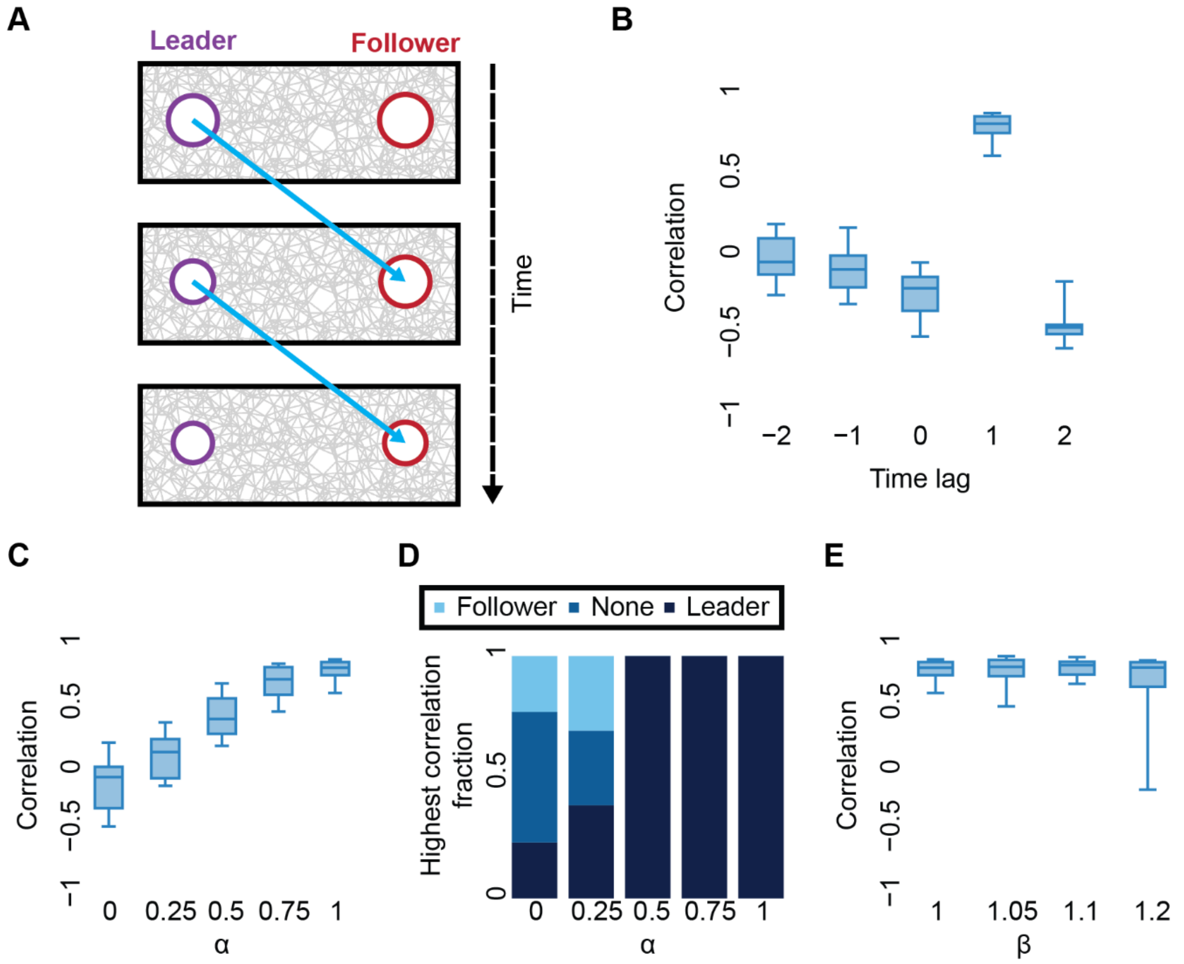

Figure S16. Identifying leader-follower relation in communicating cell pairs. (**A**) Schematic sketch of the simulation. Right cell (red, follower) mimics the contraction of the left cell (purple, leader) at the previous time point (Methods). (**B-E**) Simulated cell pair cross correlation for different time lags. N = 13 cell pairs. Pair distance = 7 cell diameters. The leader cell draws its contraction in each step independently from a normal distribution with mean of 1% and standard deviation of 0.5%. The follower cell draws its “intrinsic” component (contraction follower) from the same distribution. The second derivative of the fiber density dynamics was used for correlation. Time lag is applied to the follower’s time series before correlation is calculated. (**B**) Distribution of cell pair correlations as a function of the time lag. (**C-D**) Identifying leaders and followers as a function of varying values of α. Increased α values imply an increased contribution of the extrinsic component - the influence by the leader cell. (**C**) Distribution of cell pair correlation with time lag = 1 as a function of α values. (**D**) Fraction of cell pairs where the leader (time lag 1 or 2), follower (time lag -2 or -1) or none (time lag 0) lead to maximal cell pair correlation, and is thus identified as leader, as a function of α values. (**E**) Distribution of cell pair correlation with time lag = 1 as a function of β values. Increased β implies an increased contraction by the follower cell. α value was set to 1.

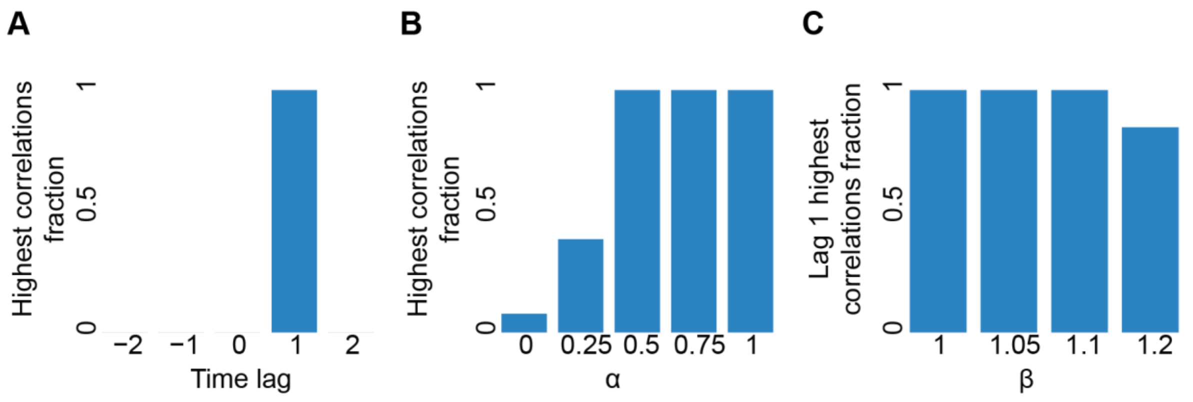

Figure S17. Leader-follower lag time analysis in simulated cell pairs. Cell pair cross correlation for different time lags. N = 13 cell pairs. Pairs distance = 7 cell diameters. The leader cell draws its contraction in each step independently from a normal distribution with mean of 1% and standard deviation of 0.5%. The follower cell draws its "intrinsic" component (contraction follower) from the same distribution. The second derivative of the fiber density dynamics was used for correlation. Time lag is applied to the follower's time series before correlation is calculated. (**A**) Distribution of time lags that lead to maximal cell pair correlation. (**B**) Fraction of cell pairs where the simulated time lag (that was set to one step) leads to maximal cell pair correlation as a function of α values. Increased α implies an increased contribution of the extrinsic component - the influence by the leader cell. Note that α = 0 implies no leader effect and thus the fraction is expected to be random. (**C**) Fraction of cell pairs where the simulated time lag leads to maximal cell pair correlation as a function of β values. Increased β implies an increased contraction by the follower cell. α value was set to 1.

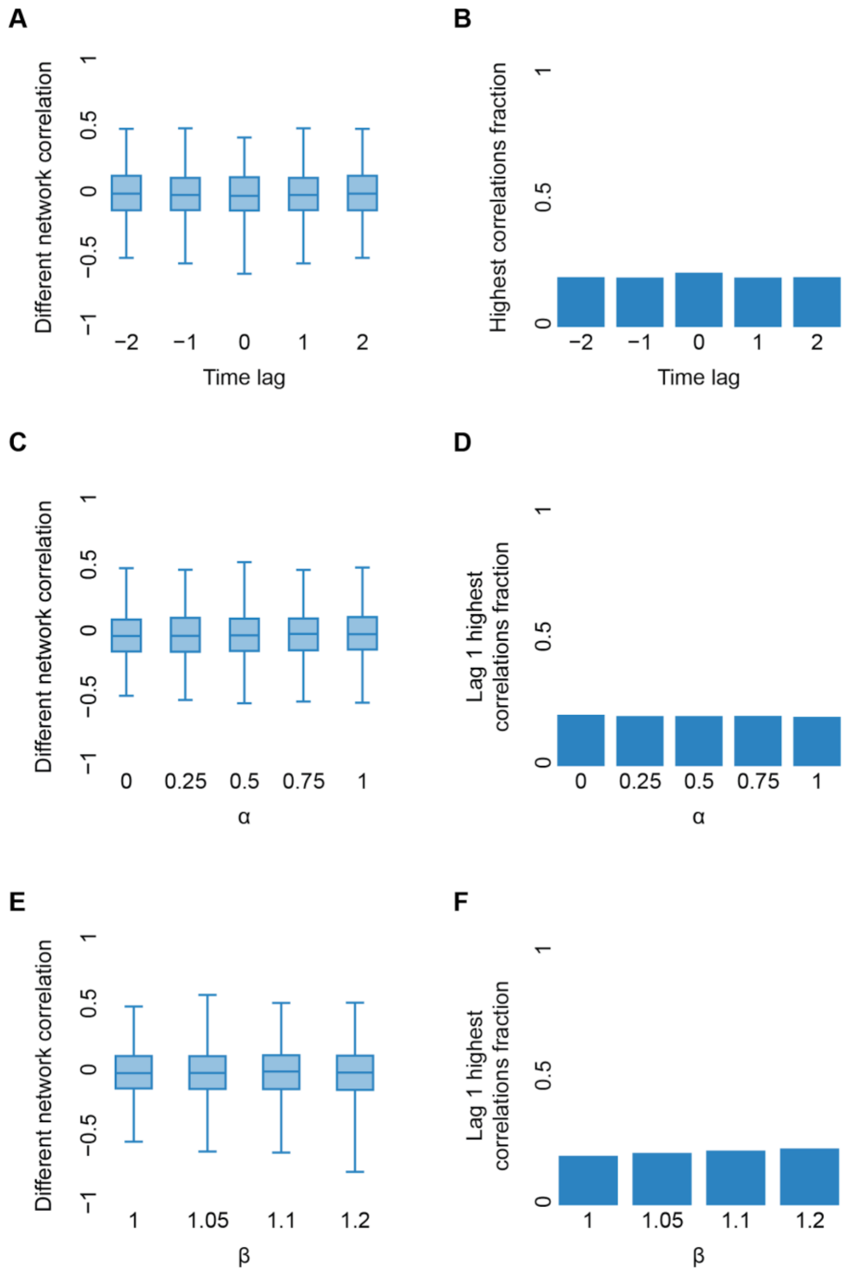

Figure S18. Cross correlation analysis of artificial pairing of simulated leader/follower cells that did not interact with one another as a control. Cross correlation for different time lags between a cell from one communicating pair and another cell from a different communicating pair. N = 13 cell pairs, pair distance = 7 cell diameters. The leader cell draws its contraction in each step independently from a normal distribution with mean of 1% and standard deviation of 0.5%. The follower cell draws its "intrinsic" component (contractionfollower) from the same distribution. The second derivative of the fiber density dynamics was used for correlation. The time lag is applied to the follower's time series before the correlation was calculated. (**A**) Distribution of correlations as a function of the time lag. (**B**) Distribution of time lags that lead to maximal correlation. (**C**) Distribution of the correlation for time lag = 1 as a function of α values. Increased α implies an increased contribution of the extrinsic component for the communicating cell paris before the artificial pairing to cells that never communicated with one another. (**D**) Fraction of cell pairs where the simulated time lag (that was set to one step) leads to maximal correlation as a function of α values. (**E**) Distribution of the correlation for time lag = 1 as a function of β values. Increased β implies an increased contraction by the follower

cell. α value was set to 1. (**F**) Fraction of cell pairs where the simulated time lag leads to maximal correlation as a function of β values.

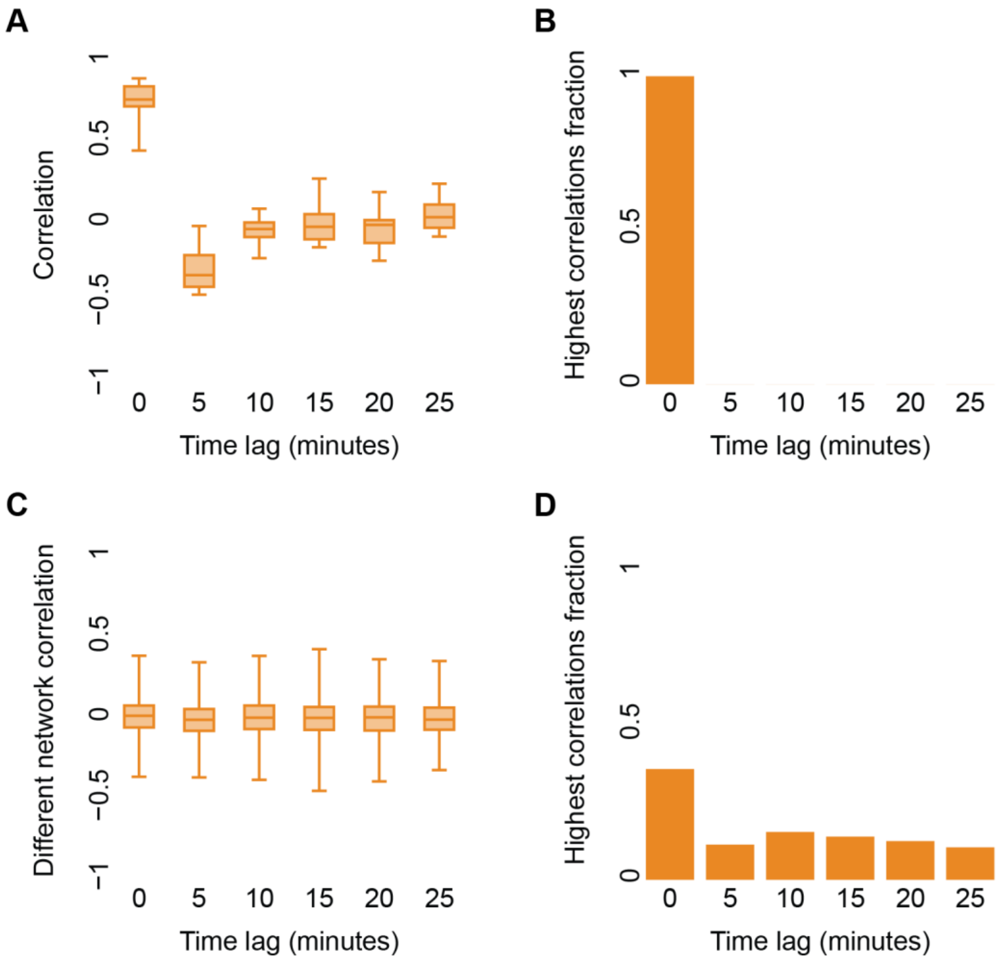

Figure S19. Cross correlation analysis did not identify leader-follower relations in experimental data. N = 19 communicating cell pairs with visible bands. Temporal resolution = 5 minutes between consecutive time frames. Pair distance of 60-150 μm. Quantification windows were placed 7.5 μm above the connecting axis between the cells. Correlations were calculated using the first derivative of fiber density dynamics. Time lags were recorded with their absolute value (symmetry argument: no preference to a specific cell as in the simulations). (**A-B**) Cell pair cross correlation for different time lags. (**A**) Distribution of cell pair correlations as a function of the time lag. (**B**) Distribution of time lags that lead to maximal cell pair correlation. (**C-D**) Cross correlation for different time lags between one cell from a communicating pair and another cell from a different communicating pair as a control. (**C**) Distribution of correlations as a function of the time lag. (**D**) Distribution of time lags that lead to maximal correlation.

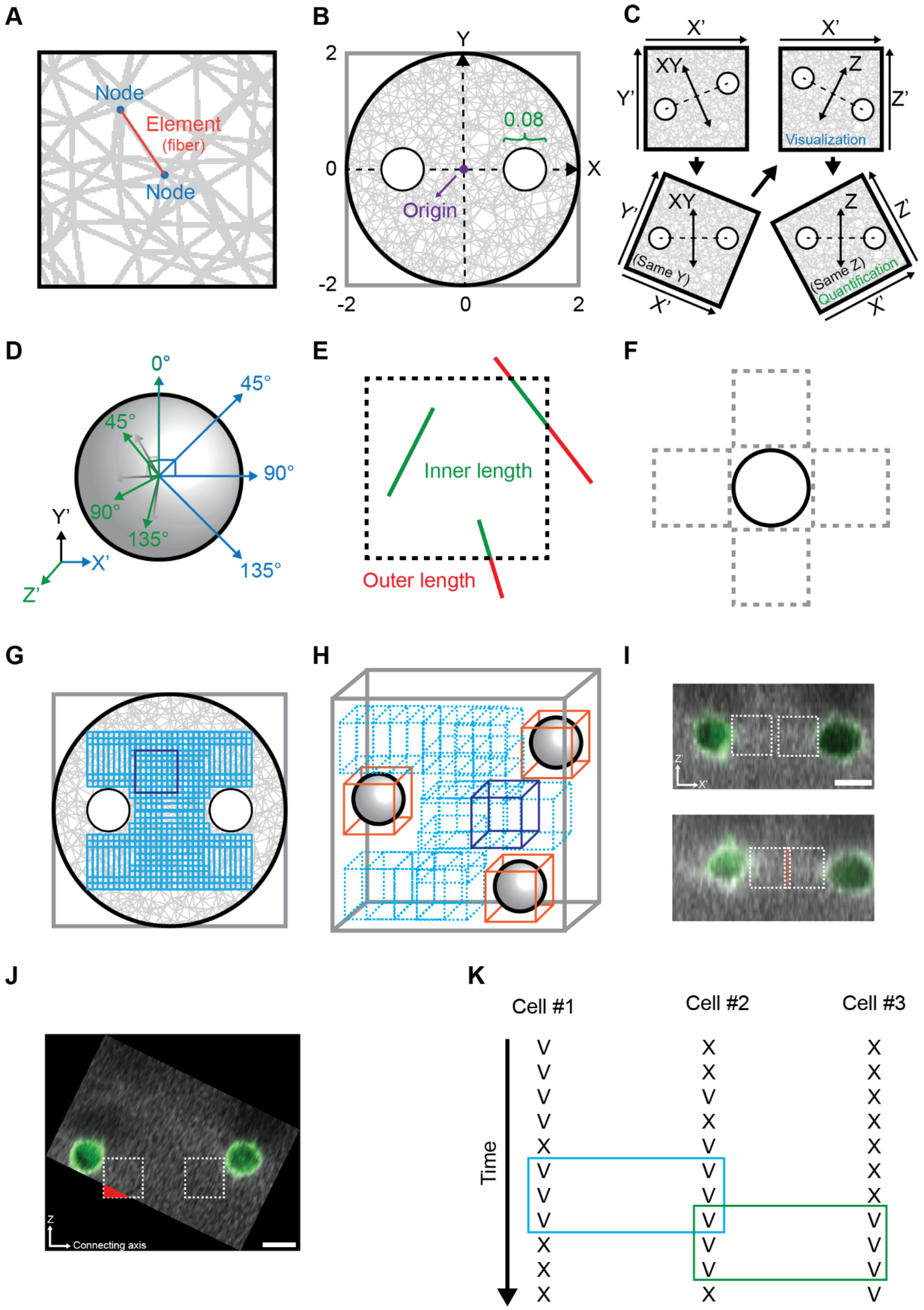

Figure S20. Methods. (**A**) Components of a finite-element simulation network. Fibers are represented by discrete one-dimensional elements (red) connected by two nodes (blue). (**B**) Finite-element network dimensions. The simulated fibrous network was defined as a circular arena, of radius 2 AU,

that is composed of connected elements. One or two circular voids, of radius 0.08 AU (green), define the cell/s. The center of the network is called the origin (purple). Single cells centers are located in the origin. Cell pairs centers are located along the X axis in predefined distance from the origin. Ratio of cell radius and the network radius in the depiction does not resemble the actual proportion between the two. (**C**) Transformation of cell pairs images. First transformation (top-left to bottom-left) aligns the cell centers to a common Y'-axis. Images are in top-view perspective. We use X' and Z' axes for cell pair visualization (top-right). The second transformation (top-right to bottom-right) aligns the cell centers to a common Z'-axis. Images are in side-view perspective. The transformed image (bottom-right) is used for cell pair quantification. (**D**) Transformation of single cell images. Illustration is in top-view perspective. First transformation (blue) in 0°, 45°, 90° or 135° in the XY axis. Second transformation (green) in 0°, 45°, 90° or 135° in the Z' axis. (**E**) Quantifying fiber (simulation elements) intersection with the quantification windows in simulations. The quantification window is illustrated with a dashed box. To be considered, a fiber must be either exclusively inside the quantification window or crossing the window's boundaries. The sub-fiber within the window's boundaries (green) are included while sub-fibers external to the quantification window (red) are excluded from the quantification. (**F**) The quantification windows for simulated single cells are placed in four directions at 90 degrees radial intervals. (**G**) Quantification windows in simulations used for computing the normalization parameters. Quantification window (blue as an example) is the size of a cell diameter by a cell diameter (0.08 x 0.08 AU). All quantification windows used for this computation (cyan) are located inside the network boundaries and outside the cells with a step resolution (overlap) of 0.02 (AU) in each of the axes. (**H**) Quantification windows in experiments used for computing the normalization parameters. Quantification window (blue as an example) length is 15 μm, approximately the cell diameter in 3D. All quantification windows used for this computation (cyan) are located exclusively inside the image boundaries in all axes and without intersection with bounding boxes (orange) around the cells, with a step resolution (overlap) of 1/10 of the image axis length for each axis. (**I**) An example of overlapping quantification windows between a pair of communicating cells. Images are in a side-view average projection perspective. Quantification windows are marked in white dashed boxes. At the onset of imaging (top) the quantification did not overlap, however after 3.5 hours of live cell imaging (bottom) the quantification windows did overlap (orange area). Scale bar = 15 μm. (**J**) An example of a quantification window extending beyond the image boundaries at the onset of imaging. Image is in a side-view perspective with averaged pixel intensities visualization after the transformation. Quantification windows are marked in white dashed boxes. Red area represents the sub-window outside the image boundaries. Scale bar = 15 μm. (**K**) Valid ("V") and invalid ("X") quantification windows (see Methods for definition) over time of three different cells. Blue rectangle represents the longest shared valid temporal sequence for cell #1 and cell #2 and the green rectangle for cell #2 and cell #3.

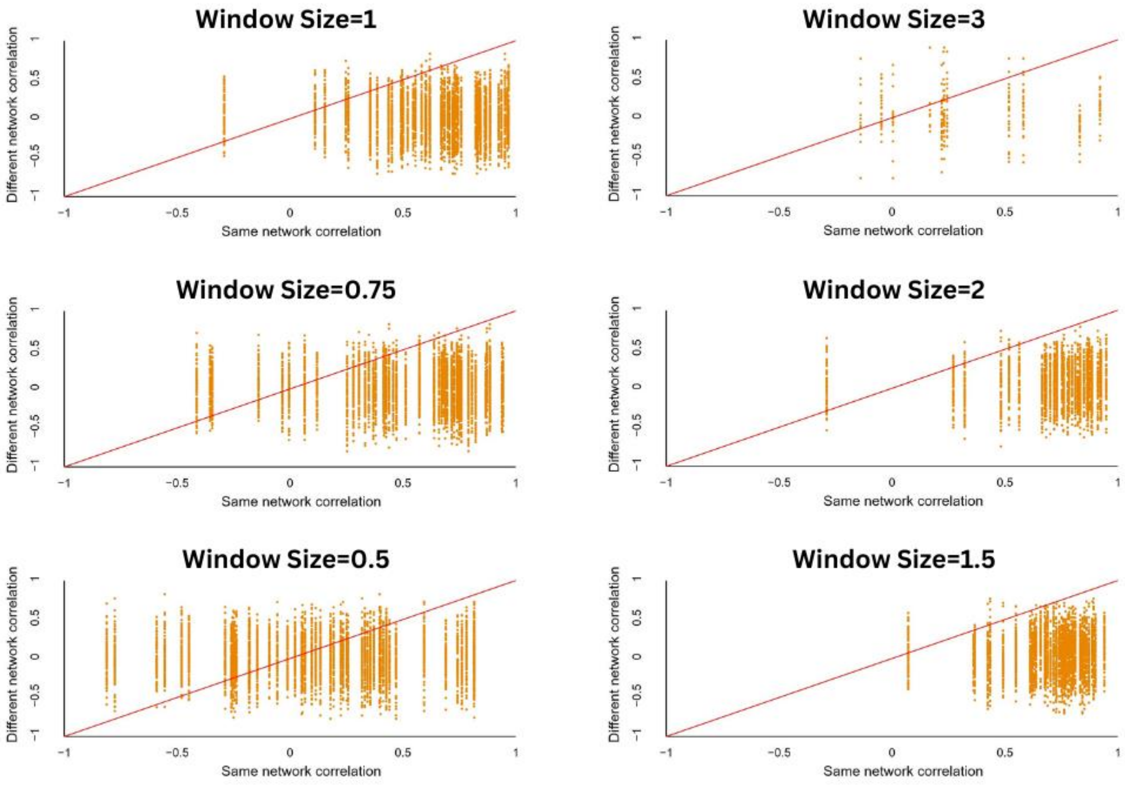

Figure S21. Sensitivity analysis for the size of the quantification. Same-versus-different pairs analysis for window size of 0.5-3 cell diameters. We selected window size of 1-cell diameters because it balanced same-versus-different performance and the number of cell pairs that can be analyzed (larger windows reduces the number of cell pairs due to overlap). Window size = 0.5: N=51 pairs, "same" pair had higher correlation in 60.9% matched correlations. Window size = 0.75, N=51 pairs, "same" pair had higher correlation in 86.7% matched correlations. Window size = 1, N=48 pairs, "same" pair had higher correlation in 94.4% matched correlations. Window size = 1.5, N=44 pairs, "same" pair had higher correlation in 98.5% matched correlations. Window size = 2, N=36 pairs, "same" pair had higher correlation in 96.5% matched correlations. Window size = 3, N=12 pairs, "same" pair had higher correlation in 76.5% matched correlations.

Supplementary tables legends

Table S1: Summary of experimental results, measuring cell-ECM-cell communication in different cell systems, imaging and experimental conditions. Same-versus-different, real-versus-fake and matchmaking results were recorded for each condition.

Table S2: Simulation data table. These data include single cells and cell pairs, heterogeneity and leader / follower in pair distances 5, 7 and 9 cell diameters.

Table S3: Experimental data table. Experimental data was partitioned to different categories as followed. (1) Whether the experiment involved single cell or cell pairs, (2) Which cell line (fibroblasts/cancer) or beads were used, (3) Whether cells were treated with Blebbistatin or whether the (control) experiment included dead cells, (4) Are the cells “real” or is it a “fake” cells analysis, (5) Whether the cell pairs had a dense fibrous band between them, (6) What was the temporal resolution (in minutes), (7) What was the pair distance between the cells (4-6, 6-8 and 8-10 cell diameters, where cell diameter is estimated at ~15μm), (8) What was the Blebbistatin dosage (in nM), (9) Whether the experiment consisted exclusively dead cells, (10) What was the number (N) of observations (mostly cell pairs) in the category.

Supplementary video legends

Video S1: Finite-element simulation of a cell pair contracting in a 2D fibrous network. Pair distance = 7 cell diameters. Both cell contracts 1% for 50 steps (Methods).

Video S2: 3D representation of a cell pair quantification axes. Microscopy axes in the left bottom corner: X’ (dark green), Y’ (orange) and Z’ (cyan). Cells (green) are in different Z’ coordinates. Connecting axis (black) is between the cell’s centers. Quantification windows (yellow) are adjacent to each cell along the connecting axis (black) between the cell’s centers, left and right sides are parallel to the microscopy axial plane. The Z-axis (cyan) and the XY-axis (purple) are perpendicular to each other and to the connecting axis (black). The video demonstrates offsets between -1 and 1 cell diameters of the quantification windows in the Z (cyan) and XY (purple) axes.

Video S3: Time-lapse confocal imaging of a single fibroblast embedded in a 3D fibrin gel. XY/Z average projection visualization of a single cell (Methods). Time resolution: 21 minutes. Scale bar = 15 μm.

Video S4: Time-lapse confocal imaging of a pair of fibroblast cells embedded in a 3D fibrin gel forming a band of dense fibers in the connecting axis between the cells. XY/Z average projection visualization of a pair of cells (Methods). Pair distance: 117 μm. Time resolution: 15 minutes. Scale bar = 15 μm.

Video S5: Matchmaking between communication partners. Images are in a side-view perspective. Cell pairs are with visible bands. Quantification windows (white dashed rectangles) are adjacent to each cell along the connecting axis (black) between the cell’s centers. The video demonstrates the analysis of a cell by correlating it’s ECM remodeling dynamics with all other candidates. Correlation results are for demonstration purpose only.

Video S6: Time-lapse confocal imaging of a pair of fibroblast cells embedded in 3D fibrin gel not forming a band of dense fibers in the connecting axis between the cells. Pair distance: 110 μm. Time resolution: 15 minutes. Scale bar = 15 μm.

Video S7: Time lapse imaging of a cell pair with a manually identified illumination artifact in several time frames that were excluded from analysis.
