# Supplementary material for: Inference of long-range cell-cell force transmission from ECM remodeling fluctuations
Source: Commun Biol. 2023 Aug 3;6:811. doi: 10.1038/s42003-023-05179-1 (PMC10400639; doi:10.1038/s42003-023-05179-1)
Supplement: Supplementary file 3 — Description of Additional Supplementary Data [file 42003_2023_5179_MOESM3_ESM.docx]

**Description of Additional Supplementary Files**

**File name:** Table S1

**Description:** Summary of experimental results.

**File name:** Table S2

**Description:** Simulation data table.

**File name:** Table S3

**Description:** Experimental data table.

**File name:** Video S1

**Description:** Finite- element simulation of a cell pair contracting in a 2D fibrous network.

**File name:** Video S2

**Description:** 3D representation of a cell pair quantification axes.

**File name:** Video S3

**Description:** Time-lapse confocal imaging of a single fibroblast embedded in a 3D fibrin gel.

**File name:** Video S4

**Description:** Time-lapse confocal imaging of a pair of fibroblast cells forming a band.

**File name:** Video S5

**Description:** Matchmaking between communication partners.

**File name:** Video S6

**Description:** Time- lapse confocal imaging of a pair of fibroblast cells not forming a band.

**File name:** Video S7

**Description:** Time lapse imaging of a cell pair with a manually identified illumination artifact.
